# Predictive Capacity of COVID-19 Test Positivity Rate

**DOI:** 10.3390/s21072435

**Published:** 2021-04-01

**Authors:** Livio Fenga, Mauro Gaspari

**Affiliations:** 1 Italian National Institute of Statistics, 00184 Roma, Italy; fenga@istat.it; 2 Department of Computer Science and Engineering, University of Bologna, 40126 Bologna, Italy

**Keywords:** COVID-19, test positivity rate, predictive capacity, health system management

## Abstract

COVID-19 infections can spread silently, due to the simultaneous presence of significant numbers of both critical and asymptomatic to mild cases. While, for the former reliable data are available (in the form of number of hospitalization and/or beds in intensive care units), this is not the case of the latter. Hence, analytical tools designed to generate reliable forecast and future scenarios, should be implemented to help decision-makers to plan ahead (e.g., medical structures and equipment). Previous work of one of the authors shows that an alternative formulation of the Test Positivity Rate (TPR), i.e., the proportion of the number of persons tested positive in a given day, exhibits a strong correlation with the number of patients admitted in hospitals and intensive care units. In this paper, we investigate the lagged correlation structure between the newly defined TPR and the hospitalized people time series, exploiting a rigorous statistical model, the Seasonal Auto Regressive Moving Average (SARIMA). The rigorous analytical framework chosen, i.e., the stochastic processes theory, allowed for a reliable forecasting about 12 days ahead of those quantities. The proposed approach would also allow decision-makers to forecast the number of beds in hospitals and intensive care units needed 12 days ahead. The obtained results show that a standardized TPR index is a valuable metric to monitor the growth of the COVID-19 epidemic. The index can be computed on daily basis and it is probably one of the best forecasting tools available today for predicting hospital and intensive care units overload, being an optimal compromise between simplicity of calculation and accuracy.

## 1. Introduction

One of the aspects that makes the COVID-19 pandemic difficult to control, is the simultaneous presence of significant numbers of both critical and asymptomatic to mild cases. While, for the former reliable data are available (in the form of number of hospitalizations and/or beds in Intensive Care Units (ICUs)), this is not the case of the latter [1,2,3]. In many instances, in fact, those who contracted the virus are unaware of such a condition and, thus, enter the status of spreaders or, in the worse case, superspreaders. Such a phenomenon, commonly referred to as under-ascertainment, is the primary reason for a disease to spread uncontrolled. Should it be not carefully checked nor effectively counteracted, it can potentially grow indefinitely, posing severe health problems at a global level and severely impacting whole health systems. Action-wise, such a situation calls for at least two measures: on the one hand, policy- and decision-makers should plan ahead the needs in terms of medical structures and equipment, whereas, on the other hand, analytical tools designed to generate reliable forecast and future scenarios should be implemented. While a number of effective approaches have been studied and proposed for different epidemics over the years, this is not the case of the COVID-19 pandemic. In fact, all the efforts so far done to model and predict such a disease might hardly support the idea that a uniformly “better” model is available to describe and predict the evolution of such a catastrophic pandemic. Therefore, even though many valid contributions have been proposed so far [4], it is not unreasonable to look at those efforts as the building block of one or more best practices. In particular, the forecasting problem has been addressed for two of the the most populated countries in the world, i.e., China [5] and India [6]. A survey including other approaches is presented here [7]. The complexity of such a task is discussed in Reference [8], where the authors analyzed three different regional-scale models for forecasting and assessing the course of the pandemic. Along those lines, it is worth mentioning the excellent article [9], where the main reasons leading to the failure of forecasting models are presented. Finally, two different predictive approaches have been proposed for Italy, i.e., one exploiting the bootstrapped prediction generated by a model of the type ARMA [10] and one based on the simulated annealing algorithm [11].

The Test Positivity Rate (TPR) is one of the indexes often used worldwide for monitoring the progression of the COVID-19 pandemic; see, for example, the coronavirus testing dataset [12], which contains an updated picture of the international situation concerning testing strategies and the associated data for many countries. Until now, the TPR was mainly studied considering its relationship with confirmed cases [13]; for example, it was used to estimate COVID-19 prevalence in the different states of U.S. [14]. However, a more intensive use of diagnosis tests associated with a standardization of the TPR, crucial in light of differences in the available tests, can solve their limited investigation abilities (see, e.g., Reference [15]).

In more details, a recent work of one of the authors [16] shows that a standardized COVID-19 Test Positivity Rate (TPR) can be used to predict hospital overload. In particular, by observing its trend, it is possible to forecast the course of patients admitted in hospital and in intensive care units. For example, when the TPR reaches a peak, a growth in COVID-19 hospitalizations lasting 12–15 days can be inferred.

There is an intuitive motivation behind such a behavior: COVID-19 epidemiological data show that symptoms, on average, occur 11 days after the contraction of the infection and that critical patients are admitted in the hospital about 4 days later. If we assume that the TPR is a measurement of the infections occurring in a given day, in an ideal situation, the infected people with a critical evolution will be presumably admitted in hospitals 15 days later. More precisely, if the TPR increases in a given day, an increasing number of active cases (including the unknown ones) can be inferred for the same day, and presumably the number of infections is increasing too. Thus, after a while the number of hospitalized people will also increase. In other words, the insight is that the TPR index models the trend of the COVID-19 infections, and it is designed to embody the unknown cases. Clearly, for this measure to be valid, all the administered diagnostic tests should be considered in the TPR calculation, as pointed out in Reference [16]. However, there are known biases involving diagnostic tests data that are difficult to deal with, e.g., those related to reporting delays [12]. As a result, the ideal predictive capacity cannot be assumed in practice, especially if different kinds of tests are used, as in the case of the current Italian situation.

**Figure 1:**
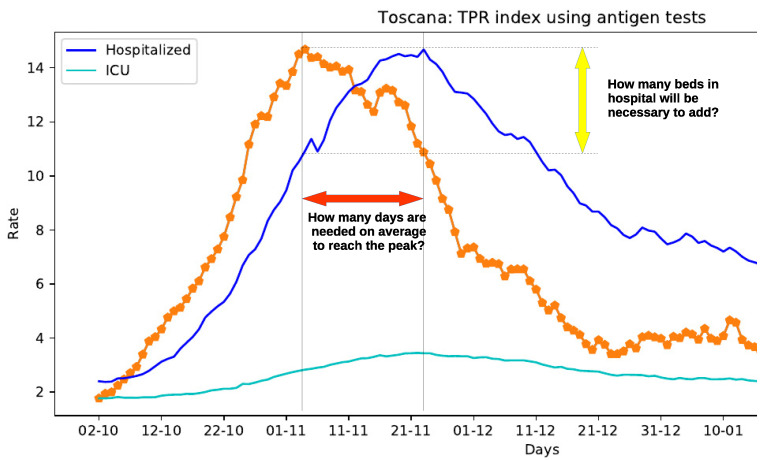
The Test Positivity Rate (TPR) index (orange dotted line) predictive capacity.

Despite these limitations, the TPR defined in Reference [16] can be effectively used to deduct important information on the course of the disease, as illustrated in Figure 1, where the epidemic course in Toscana region in autumn 2020 is depicted. This figure also plots the time series of patients admitted in hospitals and in intensive care units. An interesting correlation between the curves can be observed: the TPR peak anticipates the peak of patients admitted in hospitals and intensive care units.

The aim of this research is to analyze in details similar scenarios, especially when the TPR is growing considerably, to get to the heart of some hard-hitting questions, left as future work in Reference [16]: How many days will be needed to reach the peak of hospitalized people? How many beds in hospitals will be necessary to add? And, in general, which is the “theoretical” predictive capacity of the TPR index defined in Reference [16]?

Starting from this motivation, we analyzed the TPR index time series, as well as the hospitalized, and ICU patients time series, to investigate the predictive capacity of the TPR index, e.g., to individuate the time lags that can be effectively inferred from the available data. We first introduce the statistical methodology used, and then we present a detailed analysis for four Italian regions, for which data on antigen tests were available as reported in Reference [16].

The lagged correlation between the TPR and hospitalized people time series will be modeled using a rigorous statistical model, i.e., of the type SARIMA (short for Seasonal Auto Regressive Moving Average). A generalization of the ARIMA (Auto Regressive Moving Average) class [17], SARIMA models have been introduced to model complex dynamics of the type stochastic seasonal in many fields of research, such as economics [18,19], engineering [20], or hydrology [21]. In epidemiology, *SARIMA* models have been applied in a variety of studies: in Reference [22] the authors applied this model for estimating case occurrence of two diseases: malaria and hepatitis A from January 1980 to June 1995 for the United States, whereas, in Reference [23], the epidemiological and etiological characteristics of influenza have been identified by establishing suitable SARIMA models. In particular, such an approach proved to be accurate in the forecasting of the percentage of visits for influenza-like illness in urban and rural areas of Shenyang (China). More recently, Reference [24] used the SARIMA method—in conjunction with models belonging to the class exponential smoothing—to predict the trend of acute hemorrhagic conjunctivitis disease and used the obtained outcomes to provide evidence for the government to formulate policies regarding its prevention in mainland China.

The proposed mathematical model allowed us to estimate a predictive lag of about 12 days of the TPR for the prediction of hospitalized people time series in some Italian regions. Moreover, we defined a methodology to forecast the number of beds in hospitals and intensive care units needed 12 days ahead. The obtained results show that a standardized TPR index is a valuable metric to monitor the growth of the COVID-19 epidemic. The index can be computed daily and it is probably one of the best forecasting tools available today for monitoring hospital and intensive care units overload, being an optimal compromise between simplicity of calculation and accuracy.

## 2. Material and Methods

The TPR is one of the metrics commonly used to infer the level of transmission of a disease in a population [25] and, as such, has been also used in the case of the COVID-19 for different purposes; see, for example, References [12,14,26]. However when different types of tests are used, as it happened during the second phase of the pandemic in Italy, where antigen tests have been extensively used, the definition of the TPR becomes more critical. In this study, we will use the basic standardized version of the TPR index defined by one of the authors [16], which allows to integrate antigen tests in the index calculation.

Following the style of Reference [16], where the Greek letters Θ, Φ, and μ have been replaced, respectively, with the letters τ, ρ, and ω, for consistency with the statistic notation later employed, the mean TPR index τ on ω days is defined as follows:

(1) $$\tau_{\omega }^{A}=\frac{{\bar{dayP}}_{\omega }\times 100}{{\bar{dayT}}_{\omega }+{\bar{dayA}}_{\omega }-\bar{dayR_{\omega }}-Pr},$$

where ${\bar{dayP}}_{\omega }$, ${\bar{dayT}}_{\omega }$, and ${\bar{dayA}}_{\omega }$ are, respectively, the average values of new positive cases, molecular (PCR) tests, and antigen tests done in the last ω days. To compute the TPR index, the average number of healed patients in the last ω days, ${\bar{dayR}}_{\omega }$ and an estimation for the number of repeated tests Pr are subtracted from the total number of tests. We assume that at least one test is done for each healed patient. The number of repeated tests Pr is computed using the Equation (2), following the approach presented in Reference [16]:

(2) $$Pr=\frac{{\bar{dayA}}_{\omega }\times {\bar{dayP}}_{\omega }}{\bar{dayT_{\omega }}+{\bar{dayA}}_{\omega }}.$$

This equation is obtained assuming that the positivity rates for antigen tests and molecular tests are the same, and, thus, dayA/Pr = dayT/(dayP−Pr). Using this approach, the computed Pr can be considered an upper bound because the molecular tests positivity rate is generally greater then the one related to antigen tests, which are mainly used for screening purposes; see, for example, Reference [27].

Finally, following the style of Reference [16], a factor ρ is added to τ in order to model the impact of the number of tests on the remaining susceptible individuals, which are computed removing the total infected cases *I* from the population *N* of a given region. The number of tests are subtracted removing the repeated ones and those used for healed patients, obtaining the following formula:

(3) $$\rho =\frac{N-I-{\bar{dayT}}_{\omega }-{\bar{dayA}}_{\omega }+\bar{dayR_{\omega }}+Pr}{N-I},$$

and the TPR index $\tau_{\omega }$ is defined as follows:

(4) $$\tau_{\omega }=\tau_{\omega }^{A}\times \rho .$$

### 2.1. The Statistical Method Applied

Throughout the paper, the time series of interest, say $x_{t}$, is always intended to be a real–valued, uniformly sampled, sequence of data points of length *T*, formally expressed as

(5) $$x_{t}:=\left\{{\left(x_{t}\right)}_{t\in Z^{+}}^{T}\right\}.$$

Furthermore, $x_{t}$ is supposed to be a realization of an underlying stochastic process of the type SARIMA (short for Seasonal Auto Regressive Moving Average).

Mathematically, SARIMA models take the form of a *t*-indexed difference equation—being *t* as defined in Equation (5)—, i.e.,

(6) $$\phi_{p}\left(B\right)\Phi_{P}\left(B^{S}\right){▽}_{S}^{D}{▽}^{d}\left(x_{t}-\mu \right)=\theta_{q}\left(B\right)\Theta_{Q}\left(B^{S}\right)\alpha_{t}.$$

Denoting with *B*, *d*, and *D* the backward shift operator and the non-seasonal and seasonal difference operator, respectively, defining ${▽}^{d}=1-B^{d}$ and ${▽}^{D}=1-B^{D}$, we have $\phi_{p}\left(B\right)=1-\phi_{1}B-\phi_{2}B^{2}-.\ldots -\phi_{p}B^{p}$, $\theta_{q}\left(B\right)=1-\theta_{1}B-\theta_{2}B^{2}-.\ldots -\theta_{q}B^{p}$, $\Phi_{P}\left(B^{S}\right)=1-\Phi_{1}B^{S}-\Phi_{2}B^{2S}-.\ldots -\Phi_{P}B^{PS}$, and $\Theta_{Q}\left(B^{S}\right)=1-\Theta_{1}B^{S}-\Theta_{2}B^{2S}-.\ldots -\Theta_{q}B^{QS}$. Here, ϕ, θ, Φ, Θ, respectively, denote the non-seasonal autoregressive and moving average parameters and the seasonal autoregressive and moving average parameters. Finally, $\alpha_{t}$ is a 0—mean white noise with finite variance $\sigma^{2}$. In the present paper, external information is exploited and embodied in Equation (6) in the form of a matrix of regressors $D_{j,t-k}$, with $k\in Z^{+}$, weighted by a vector of coefficients $\beta_{j}$, i.e.,

(7) $$\phi_{p}\left(B\right)\Phi_{P}\left(B^{S}\right){▽}_{S}^{D}{▽}^{d}\left(x_{t}-\mu -\sum_{j=1}^{s}\beta_{j}D_{j,t-k}+u_{t}\right)=\theta_{0}+\theta_{q}\left(B\right)\Theta_{Q}\left(B^{S}\right)\alpha_{t}.$$

This particular extension is usually referred to as REG−SARIMA, to stress the role played by the possibly lagged (of an amount equals to *k* temporal lags) regressors, stored in the matrix $D_{j,t}$. These types of models are designed to capture the stochastic dynamics generated by the residuals obtained by regressing the matrix *D* (the independent variable(s)) on the time series of interest (the dependent variable). A better insight of the stochastic mechanism governing the REG−SARIMA equation can be gained by re-expressing Equation (6) so as to emphasize the role played by the term $u_{t}$ in Equation (7), i.e.,

(8) $$x_{t}-\mu -\sum_{j=1}^{s}{\hat{\beta }}_{j}D_{j,t-k}∼SARIMA\left(p,d,q,P,D,Q\right).$$

This formulation makes clear the flexibility of this approach, which allows the extraction of the significant lags at which the different regressors impact the time series of interest, as well as their magnitudes.

If the integration constants *d* and *D* (introduced in Equation (8)) are certainly useful to mitigate—if not solve altogether—many stationarity problems, on the other hand they might not be effective against non-normality and/or eteroschedasticity issues. Unfortunately, the data considered in this paper are affected by both these phenomena; therefore, as a coping mechanism, the well-known one–parameter Box–Cox data transformation has been adopted. Presented in the mid-sixties in Reference [28], this method has been discussed and applied in a wide range of problems (see, among others, References [29,30,31]), given the widespread acceptance gained over the years. Its mathematical formulation is quite straightforward and takes the form of a power transformation, i.e.,

(9) $$\begin{array}{c} x_{t}\left(\lambda \right)=\left\{\begin{array}{cc} \frac{x_{t}^{\lambda }-1}{\lambda }, & \mathrm{if}\;\lambda \neq \;0\\ \ln(x_{t}), & \mathrm{if}\;\lambda =0. \end{array}\right. \end{array}$$

By embodying the λ parameter in Equation (7), the model employed in this paper is finally defined, i.e.,

(10) $$\phi_{p}\left(B\right)\Phi_{P}\left(B^{S}\right){▽}_{S}^{D}{▽}^{d}\left(x_{t}\left(\lambda \right)-\sum_{j=1}^{s}\beta_{j}D_{j,t-k}+u_{t}\right)=\theta_{0}+\theta_{q}\left(B\right)\Theta_{Q}\left(B^{S}\right)\alpha_{t}.$$

The inference procedures carried out for the estimation of Equation (10) are of two types: maximum likelihood for the SARIMA parameters $\left\{\phi ,\theta ,\Phi ,\Theta ,d,D\right\}$ and ordinary least squares for the vector β. Finally, the hyper-parameters $\left\{(p,d,q,P,D,Q)\right\}$, as well as the Box-Cox constant λ (Equation (9)), are estimated within the framework of the Information Theory as explained in the following section.

### 2.2. Estimation of the Model Order and the λ Parameter

Akaike’s Information Criterion AIC [32,33,34]—one of the most popular model selectors—will be employed to choose the SARIMA model order, as well as the Box-Cox λ parameter. The selection of those constants is not a trivial task as it entails the solution of a conditional multi-objective problem induced by the 6–dimensional vector of unknown constants $\Gamma \equiv \left\{(p,d,q,P,D,Q)\right\}$ conditional to the Box-Cox paramter λ. The estimation method employed to find the “best” conditioned vector of hyper-parameters—that is the one governing the selected order structure $M^{*}\equiv \left({\hat{\Gamma }}^{*}|\lambda^{*}\right)$—relies on the information theory and, in particular, on the Akaike Information Criterion (AIC). At its core, *AIC* is based on an estimate of the expected relative entropy (the Kullback–Leibler divergence) contained in an estimated model, that is the degree of divergence from the “true” theoretical model. Assuming $X_{t}$ to be randomly drawn from an unknown distribution H(x), with density h(x), estimation of *h* is done by means of a parametric family of distributions with densities [f(x|θ;θ∈Θ)], θ the unknown parameters’ vector. Denoting by $f(z|\hat{\theta })$ the predictive density function, by *f* the true model, and by *h* the approximating one, Kullback-Leiber divergence takes the form

(11) $$I\left(h\left(z\right);f\left(z|\hat{\theta }\right)\right)=\int h\left(z\right)\log h\left(z\right)dz-\int h\left(z\right)\log f\left(z|\hat{\theta }\right)dz,$$

which, after some algebra, can be written as follows:

(12) $$L\left(X_{n};H\right)=\int h\left(z\right)\log f\left(z|\hat{\theta }\right)dz=\int \log f\left(z|\hat{\theta }\right)dH\left(z\right).$$

This quantity can be estimated by replacing *H* with its empirical distribution $\hat{H}$, so that $L\left(X_{T};\hat{H}\right)=\frac{1}{T}\sum_{\alpha =1}^{T}\log f\left(X_{\alpha }|\hat{\theta }\right)$. This is an overestimated quantity of the expected log likelihood, given that $\hat{H}$ is closer to $\hat{\theta }$ than *H*. The related bias can be written as follows:

(13) $$b\left(H\right)=E_{H}\left\{L\left(X_{n};\hat{H}\right)-L\left(X_{n};H\right)\right\}.$$

Denoting by the Greek letter ξ, the number of estimated parameters, Akaike proved that $b\left(H\right)=\frac{\xi }{T}$, so that the information based criterion takes the form $L\left(X_{T};\hat{H}\right)+\frac{\xi }{T}$. By multiplying this quantity by −2, finally, AIC is defined as

(14) $$-2\log L(X_{T};\hat{H)}+2\xi .$$

Elaborating on Reference [35], the correct formulation of AIC for the model expressed in Equation (10) takes the form

(15) $$\begin{array}{cc} AIC\left(\Gamma |\lambda_{0}\right)= & T\log L\left(x_{t}\left(\lambda_{0}\right)|\Gamma \right)+\\ & +\left\{\frac{T}{T-(d+D)}\right\}2\left(p+q+P+Q+1+\delta_{1}+\delta_{2}\right)+T\log2\pi +T, \end{array}$$

where $\delta_{1}$ and $\delta_{2}$ are as follows (Equations (16) and (17)):

(16) $$\begin{array}{c} \delta_{1}=\left\{\begin{array}{cc} 1, & \mathrm{if}\;d=0\\ 0, & \mathrm{if}\;d\neq 0 \end{array}\right., \end{array}$$

(17) $$\begin{array}{c} \delta_{2}=\left\{\begin{array}{cc} 1, & \mathrm{if}\;D=0\\ 0, & \mathrm{if}\;D\neq 0. \end{array}\right. \end{array}$$

By sequentially applying Equation (14) for different combinations of the hyper-parameters $\left\{(p,d,q,P,D,Q))\right\}$ and conditioning the observed data to a given λ parameter (which in Equation (15) has been denoted with $\lambda_{0}$) a sequence of AIC values is obtained. This is the first of the two-step selection strategy adopted in the present paper, which is usually referred to as MAICE (short for Minimum AIC Expectation) [36] procedure. In the second step, the order $\left(\Gamma^{*}\right)$ satisfying:

(18) $$\Gamma^{*}|\lambda_{0}=\arg\min_{(\hat{\Gamma }\left|\lambda_{0}\right)\leq {\hat{\Gamma }}_{0}}AIC\left(\Gamma |\lambda_{0}\right),$$

i.e., the minimizer of the AICs generated by the candidate models, will be the winner model structure. However, Equations (15) and (18) are not designed to estimate the Box-Cox λ parameter. To this end, a grid search approach—over a set Λ of *B* competing parameters $\left\{\lambda_{j};j=1,2,\ldots ,B\right\}$—has been applied. Each λ has been evaluated in terms of the contributions given in terms of both data normalization and statistical significance of the external regressor. Finally, MAICE procedure requires the definition of an upper bound for all the Γ parameters, as a maximum order a given process can reach. This choice, unfortunately, is a priori and arbitrary.

## 3. Results

**Figure 2:**
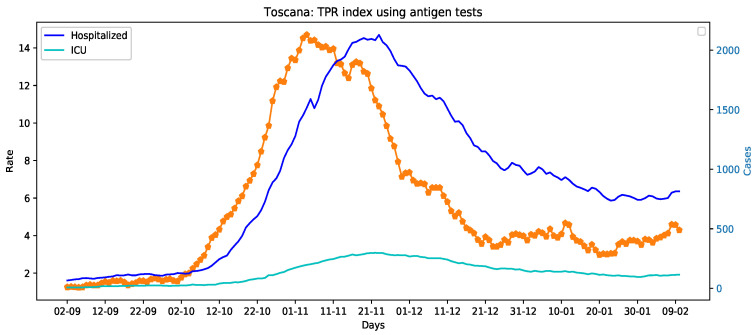

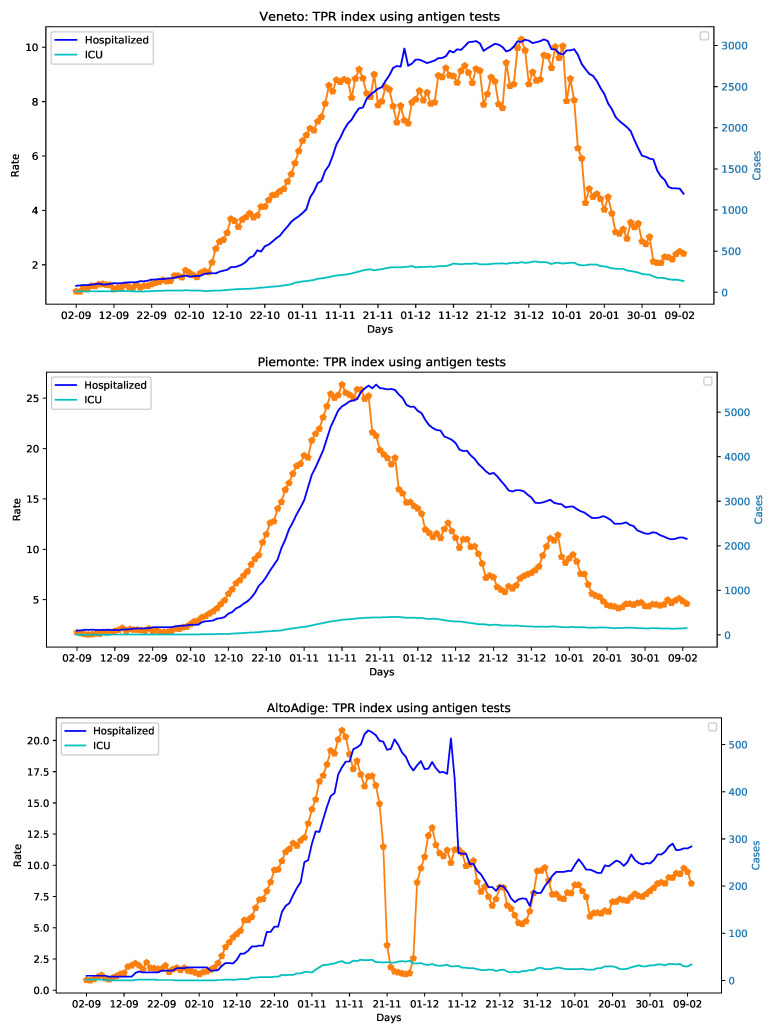
The TPR index (orange dotted lines) and hospitalized patients time series of Toscana, Veneto, Piemonte, and Alto Adige.

The data used in this paper are made available by the Italian Civil Protection Department and publicly accessible, free of charge, at the following web address: https://github.com/pcm-dpc (accessed on 11 February 2021) In more details, these data—sampled at a daily frequency—are those necessary to compute the TPR (the number of new persons tested positive for COVID-19; the number of tests done considering both molecular (PCR) tests and antigen tests, and the number of healed persons), and those related to the number of hospitalizations and beds in intensive care units occupied by patients tested positive for COVID-19. The considered time frame ranges from 2 September 2020 to 10 February 2021 for a total of 353 data points. We have analyzed 4 Italian regions for which the collection of the data on the antigen-based tests administered from October 2020 to the 15 January 2021, has been possible, i.e., Toscana, Veneto, Piemonte, and Alto Adige. The interested reader may refer to Reference [16] for the details of the data collection procedure. Unfortunately, certain data concerning the use of diagnosis tests in the considered time frame are still not available for the other Italian regions. Figure 2 presents the TPR and hospitalized time series for Toscana, Veneto, Piemonte, and Alto Adige.

The presented empirical experiment considers two different scenarios, according to the way the available information is used. Their aim is to answer the hard-hitting questions that we have set in Figure 1. The first one—which can be defined of the type real–life—exploits the whole data set and it is designed to analyze the predictive capacity of TPR, to deliver a “theoretical” time lag between the two series, and prediction which, by design, cannot be verified being projected into the unknown future. On the contrary, the second experiment concerns forecasting the number of beds needed in hospitals and intensive care units after the determined time lag in specific situations in the past, that can be verified using the available data.

### 3.1. Analysis of the TPR Predictive Capacity

In essence, this part of the experiment, being based on the whole data set, can support only qualitative considerations on the proposed method. In accordance with the intuition that TPR represents the evolution of infections, the TPR should impact the hospitalization time series 15 days in advance. Studying the lagged correlations between the TPR time series and those of patients admitted in hospitals and ICUs, using the SARIMA model, we have individuated a predictive time lag of about 12 days for all the analyzed regions, which confirm our intuitive hypothesis. Indeed, a 12 days predictive capacity for the TPR, with respect to hospitalized patients instead of the hypothesized 15, can be reasonably expected considering the above mentioned retrospective revisions effect [12]. In Table 1, we will report the time lag estimated for each region, along with an approximated multiplier accounting for the positive (negative) variation in the number of beds needed for a unit increase (decrease) of the TPR index. Table 2 presents the detailed results of the SARIMA model.

As estimates of future values which are yet to realize, these predictions can be mainly exploited to make qualitative inferences. For example, in the Veneto region, if the TPR increases of one unit, the model estimates that 82 additional beds may be needed in the near future (after 12 days). As for the ICUs, we can expect 12 additional beds. Vice versa, if the TPR decreases in Veneto, a similar amount of beds should be subtracted. In the considered regions, the average variation of beds in hospital and ICUs are 63 and 16, respectively.

### 3.2. Forecasting Hospital Overload

The second scenario envisioned, has been designed to carry out a precise evaluation of the performances delivered by the proposed method. To do so, we employed a test set with the same length but different starting point, as illustrated in Table 3. In practice, both structure and parameters of each SARIMA models has been estimated on the training set (this time with different sample sizes but same starting points) and, as already mentioned, evaluated on a “unknown” portion of data. Such a quantitative evaluation has been conducted considering different scenarios on all the studied regions: two in which the TPR was growing considerably in Toscana and Alto Adige; one associated to the beginning of the “red zone” (In the three-tiered system issued in Italy to combat the spread of COVID-19, the “red zone” indicates an high-contagion-risk area where non-essential shops and markets are closed and residents are only allowed to leave their homes for work, health reasons or emergencies.) in Piemonte; one characterized by a slow growth of the TPR index in Veneto; and one associated to a fast lowering of the TPR indicator in Veneto.

As for the REG−SARIMA model, as described in the Methods section, the model order has been defined using the MAICE procedure and constraining the Box-Cox λ parameter to 0 (i.e., log—transforming the data). However, being an exhaustive search of the “best” REG−SARIMA model either unfeasible or impractical for computational reasons, the competition set has been built following the Box-Jenkins procedure, as illustrated, e.g., in Reference [17]. Almost all the parameters of the final models are statistically significant and generate a sequence of residuals which can be deemed acceptable in terms of whiteness. Most of the times, the Maximum Likelihood algorithm converged quickly, with the only exception of the Piemonte region. In this case, a “sparse” data generating process in the autoregressive part involved a lengthy estimation approach—of the type trial and error—for the definition of the “best” (in AIC sense) model’s non-seasonal structure.

As already pointed out, the adopted MAICE Equation (18) is constrained to a specific value of the Box-Cox constant, which, therefore, has been set to $\lambda_{0}=0$. As for the maximum order $\Gamma_{0}$, it has been arbitrarily chosen on a case by case basis (see the Methods section for details).

**Figure 3:**
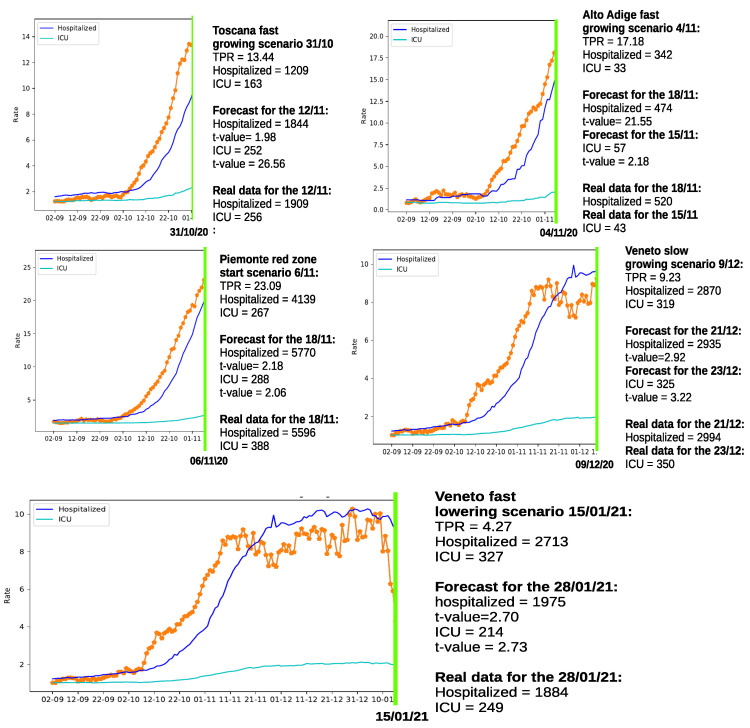
Forecasting hospitalized patients growth in 5 different scenarios for regions: Toscana, Alto Adige, Piemonte, and Veneto (also including a fast lowering example). The orange dotted lines represent the TPR index.

The results of the forecasting experiments are summarized in Figure 3. The reader will certainly notice that the best forecasting results are obtained in the last experiment concerning the fast TPR lowering scenario in the Veneto region, where more data are available. However, all the other examples provide reasonable results, and most importantly, when a fast growing of the TPR was present in the preceding of the cut, significant increases in hospitalizations are estimated. The determined increments are generally comparable to the generic estimations presented in Table 1.

## 4. Discussion

The proposed approach is general and can be exploited in any region/state under the condition that a set of requirements, below reported, are satisfied:

(1) The data on the antigen tests administrated are provided;
(2) The time series of new positive cases should include the daily number of new positives tested using only antigen tests;
(3) The TPR should reach a peak before the hospitalized and ICU patients reach theirs.

The third criterion captures the same effect dealt with in References [1,3]. In particular, in Reference [1], it is stated that: “the peak of the cases curves shifts when they are adjusted for under-ascertainment”. The rationale behind this idea is that the peak of unknown infections necessarily precedes the one related to the hospital admissions.

In general, when the first two requirements hold, then the 3rd one should hold, as well. Vice-versa, if this is not the case, probably other anomalies or errors occur in the data. Moreover, issues concerning tests reliability cannot be excluded a priori—especially when the ratio between hospitalized and positive cases growth considerably (e.g., due to tests specificity issues which might be related to new variants [37]). Should one or more of the above mentioned requirements be unfulfilled, the predictive properties of TPR might be affected. If this is the case, an integration effort should be made to collect the missing data, and/or correct possible errors. For example, even though requirement 2 was not met for the Alto Adige region, we were able to analyze the TPR by manually adding the missing information to the time series of the new positives [16].

At this point, it is worth to compare the TPR index with other COVID-19 key indicators, commonly used for monitoring purposes [38], to the end of assessing their predictive properties. In particular, we have chosen the following indicators, designed to measure the dynamical behavior of the infections, i.e.,

- Growth rate: positives daily variation;
- Incidence: fraction of COVID-19 positives per 100,000 individuals;
- The reproduction number $R_{t}$: number of secondary infections generated from a case at time *t*.

Table 4 shows the pure predictive capacity with respect to hospitalizations of these COVID-19 indicators, for comparison with the TPR. While the TPR can be considered as a measure of the number of infections that occur on a certain day, also accounting for unknown cases, indicators based on officially reported positive cases (e.g., incidence and growth rate), measure the variation of official cases in a given area. Assuming that critical cases are admitted into hospitals within 4 days after tested positive, such a delay can be taken as an approximate “upper bound” for their pure predictive capacity.

As for the reproduction number it has to be said that, being based only on the known (detected) cases, is not designed to capture the hidden variations generated by the (unknown) asymptomatic. For example, Italy and the UK experienced during the summer a strong reduction of the Rt values, which exhibited values below one. However, the data released at the beginning of the month of September, showed that, unfortunately, the virus did not stop spreading in summertime, and the Rt failed to properly react to the ongoing spreading situation. Thus, it is not unreasonable to assume the Rt predictive capacity to be approximately less or equal 4 days, consistently with other available indicators based on officially reported cases. Moreover, it might not be unlikely the reduction of such a prediction horizon, considering the computation time actually needed for this indicator to be released.

The impact of under-ascertainment (the ratio of confirmed cases to the true number of cases) on the reproduction number is also discussed in Reference [1], where the correlation between testing and the amount of unknown cases is investigated. In essence, the Rt—being based on the number of cases officially reported—should be expected to embody biasing components, to an extent directly proportional to the quota of unknown cases.

On the contrary, the TPR, as we have demonstrated, adds an approximate extra time of 11 days (the average number of days between the infection and symptoms’ onset) leading to a pure predictive time lag of about 15 days, and a “theoretical” one of about 12 days.

**Figure 4:**
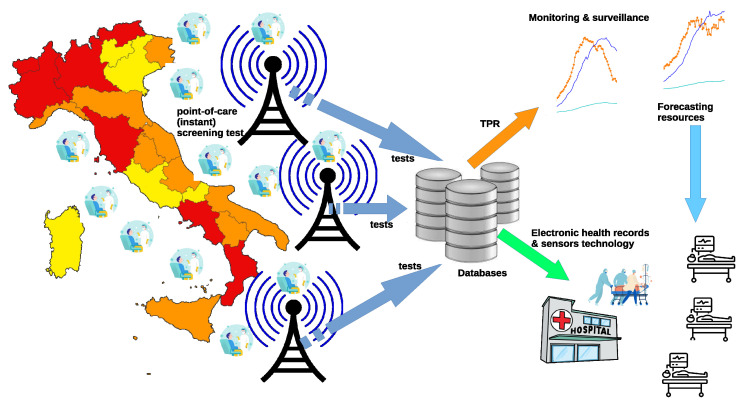
Developing point-of-care (instant) screening tests for COVID-19: data collection, sensors technology, TPR calculation, and information flows.

Last but not least, TPR precision clearly depends also on the data collection process adopted, which should be designed and implemented to guarantee the lowest possible error rates in the transmission of the test results. This also is to minimize the negative impact arising from the above mentioned retrospective revisions. Indeed, it would be possible to define more precise TPR measures provided that the data were organized in a more structured form, as discussed in Reference [16]. It is a fact that, by collecting and making available additional information—often readily available to the health care provider—the TPR would significantly improve its reliability. For example, it might be possible to gain precious insight by simply studying the effectiveness of different test typologies (diagnosis, screening, surveillance for health care operators and so forth) and associating specific accuracy information to the different types of tests administered. Clearly, the more (quality) information enter the TPR the more valuable its contribution in the description and prediction of the COVID’s dynamics. For example, data collection could be improved developing point-of-care instant screening tests [39], incorporating TPR data transmission and calculation, as depicted in Figure 4. In this scenario, an improved TPR could be fruitfully exploited for monitoring, surveillance, and forecasting purposes, as well as to integrate electronic health records with information retrieved by sensors [39]. Nevertheless, results obtained in this study emphasize the effectiveness of the proposed approach.

## 5. Conclusions

In this paper, we have presented a forecasting method for the short term prediction of the impact of COVID-19 disease on the public health system. To this end, we have provided enough evidence about the goodness of the TPR as a leading indicator for both the number of people hospitalized and, out of this group, for those who required a bed in intensive care units. The theoretical framework chosen—that is the time series analysis—has been particularly useful for the dynamic comparison and the exploitation of the information contained in the TPR time series. In our simulations, the model chosen, of the type REG−SARIMA, was able to generate reliable predictions from a minimum of 8 to 12 lags. However, especially in light of new developments of the disease—which take the form of many variants—the prediction performances of the REG−SARIMA model might be affected, if not impaired altogether. Therefore, future directions include the study of a more appropriate model, e.g., of the type regime-switching. Furthermore, additional external information (e.g., the time varying percentage of critical cases) could be fruitfully exploited in a Bayesian theoretical framework (e.g., of the type Bayesian Hidden Markov Models [40]) or using heuristic based approaches (e.g., like the Dempster–Shafer techniques [41]). Finally, we will consider the remaining Italian regions as soon as time series of “enough” length become available.

## Figures and Tables

**Table 1:** This table presents the results of the regression models with Seasonal Auto Regressive Moving Average (SARIMA) errors concerning patients admitted in hospitals and intensive care units for Toscana, Veneto, Alto Adige, and Piemonte regions. The columns Days and Beds indicate the TPR predictive capacity in days (with the associated *t*-value) and the estimated variation of beds in both hospitals and Intensive Care Units (ICUs).

Region	Days	Hospitalized *t*-Value	Beds	Days	ICU *t*-Value	Beds
Toscana	12	2.34	54	12	2.05	9
Piemonte	12	3.82	86	12	2.03	36
Veneto	13	2.07	82	13	2.52	12
Alto Adige	12	1.92	30	12	5.60	8

**Table 2:** This table presents the detailed results of the experiment presented in Section 3.1, for studying the SARIMA lagged correlation between the TPR time series and those of patients admitted in hospitals and ICUs. The last two columns Days and Beds indicate the TPR predictive capacity in days and the number of additional beds in hospital or ICU after 12 days for each TPR unit.

**Toscana**										
SARIMA(2, 1, 0)(1, 0, 1) Box Cox trans: λ = 1.2 $\beta_{t-value}$ = 2.34										
	$\phi_{1}$	$\phi_{2}$	$\Phi_{1}$	$\Theta_{1}$	β	Days	Beds			
Hosp:	0.45	0.18	0.91	−0.69	100.61	12	54			
s.e.	0.08	0.09	0.063	0.13	43.00					
SARIMA(2, 1, 0)(0, 0, 1) Box Cox trans: λ = 1.2 $\beta_{t-value}$ = 2.05										
	$\phi_{1}$	$\phi_{2}$	$\Theta_{1}$	β	Days	Beds				
ICU:	0.12	0.29	0.15	10.18	12	9				
s.e.	0.09	0.09	0.09	4.98						
**Veneto**										
SARIMA(2,1,1)(1,0,1) Box Cox trans: λ= 1.4 $\beta_{t-value}$=2.07										
	$\phi_{1}$	$\phi_{2}$	$\theta_{1}$	$\Phi_{1}$	$\Theta_{1}$	β	Days	Beds		
Hosp:	0.73	0.21	−0.76	0.77	−0.60	341.70	13	82		
s.e.	0.11	0.09	0.08	0.15	0.19	164.82				
SARIMA(0,1,1)(0,1,1) Box Cox trans: λ = 1.34 $\beta_{t-value}$ = 2.52										
	$\theta_{1}$	$\Theta_{1}$	β	Days	Beds					
ICU:	0.06	−0.67	20.72	13	12					
s.e.	0.09	0.09	8.22							
**Alto Adige**										
SARIMA(3, 1, 0)(0, 1, 1) Box Cox transf: λ = 1.69 $\beta_{t-value}$ = 1.92										
	$\phi_{1}$	$\phi_{2}$	$\phi_{3}$	$\Theta_{1}$	β	Days	Beds			
Hosp:	0.27	−0.25	0.36	−1.00	182.07	12	30			
s.e.	0.08	0.08	0.09	0.07	94.66					
SARIMA(0, 0, 3)(0, 1, 2)) Box Cox transf: λ = 1.98 $\beta_{t-value}$ = 5.60										
	$\theta_{1}$	$\theta_{2}$	$\theta_{3}$	$\Theta_{1}$	$\Theta_{2}$	β	Days	Beds		
ICU:	1.06	1.04	0.63	−0.58	−0.30	27.12	12	8		
s.e.	0.06	0.07	0.06	0.13	0.10	4.84				
**Piemonte**										
SARIMA(10,1,1)(1,1,1) Box Cox: λ = 3 $\beta_{t-value}$ = 3.82										
	$\phi_{3}$	$\phi_{5}$	$\phi_{6}$	$\phi_{10}$	$\Theta_{1}$	$\Phi_{1}$	$\theta_{1}$	β	Days	Beds
Hosp:	0.22	0.11	0.31	0.19	0.16	0.21	−1.0	211,187.31	12	86
s.e.	0.08	0.07	0.08	0.07	0.09	0.09	0.05	55,285.44		
SARIMA(3,1,0)(0,1,1) Box Cox: λ=1.2 $\beta_{t-value}$=2.03										
	$\phi_{1}$	$\phi_{2}$	$\phi_{3}$	$\theta_{1}$	β	Days	Beds			
ICU:	0.38	0.36	0.17	−0.83	60.33	12	36			
s.e.	0.08	0.08	0.08	0.08	29.74					

**Table 3:** Forecasting dates in different situations: training and test set.

Region	Situation	Training Set	Obs	Test Set	Obs
Toscana	fast growing	02/09/20–10/31/20	60	01/11/20–15/11/20	15
Piemonte	red zone start	02/09/20–06/11/20	66	07/11/20–22/11/20	15
Veneto	slow growing	02/09/20–09/12/20	99	13/12/20–28/12/20	15
Veneto	fast lowering	02/09/20–29/12/20	136	15/01/21–30/01/21	15
Alto Adige	fast growing	02/09/20–04/11/20	64	05/11/20–20/11/20	15

**Table 4:** Pure predictive capacity in days of different COVID-19 indicators with respect to hospitalization.

Metrics	What It Represents	Days
TPR	Number of active cases in a region also	15
	embodying the unknown portion of asymptomatic	
Growth rate	Variation of detected positive cases in a region	4
Incidence	Number of known cases in a region	4
$R_{t}$ index	Variation of the infections dynamics in a region	4

## Data Availability

The data used in this paper are made available by the Italian Civil Protection Department and publicly accessible, free of charge, at the following web address: https://github.com/pcm-dpc (accessed on 11 February 2021). The TPR and hospitalized time series are available at the following web address: http://www.cs.unibo.it/~gaspari/www/italy.html (accessed on 11 February 2021).
